# Urethral Stricture: Its Treatment and Prognosis

**Published:** 1904-08-20

**Authors:** 


### URETHRAL STRICTURE: ITS TREATMENT AND PROGNOSIS.

Keyes[1] believes that where a stricture is properly treated the prognosis is good, but if not properly treated it is bad. He is also of opinion that the proper treatment of the cause will prevent the occurrence of stricture. The causes are gonorrhœa and trauma. Some look upon gleet as the cause, but many cases of gleet continue for a long time and yet cause no stricture, although gleet is often the first symptom of a contracting stricture. Keyes claims that after employing the so-called "abortive treatment" for gonorrhœa for three years he has not had stricture follow gonorrhœa once. Traumatic stricture is attributable to two causes, urethral instrumentation and perineal injury. The sensitiveness of the urethra is recognised more now than it used to be, and therefore stricture from the former cause is not as often met with as it was formerly, but with all possible care it is sometimes not preventable where a catheter is being continuously passed—*e.g.* for prostatic hypertrophy—but as a rule such a stricture is superficial, soft, and readily yields to dilatation. Traumatic stricture due to perineal injury, as occurs where a patient falls astride a plank, is the most intractable and resilient type. To avoid this, the only safe procedure, even where the injury to the urethra is small, is to do an external urethrotomy and prevent dense scar formation by incising the contused canal and draining it; then any stricture which may ensue will be relatively benign and dilatable. Where a stricture is actually present the prognosis must depend upon its dilatability. If it is dilatable the surgeon will recognise the gradual absorption of the scar tissue. A stricture may be undilatable from one of four causes: It may be (1) resilient where, after each passage of the sound, it recontracts to its former size; (2) irritable where it resents the passage of the sound, showing inflammatory reaction of different forms; (3) impassable where the instrument cannot be passed through at all; (4) complicated where there is stone, extravasation, etc. Resilient strictures are met with in the penile urethra, and to cure these internal urethrotomy is necessary, followed by the occasional passage of a sound for two or three months. In the deeper urethra resilient strictures are usually traumatic. These are so dense, that nothing short of perineal section and excision of all the scar tissue will affect a cure—they should not be temporised with. Where a stricture is irritable two courses are open, the one mild, slow, and uncertain, the other radical but dangerous. If circumstances allow the former should be followed; local treatment is stopped and general treatment pushed—antiseptics as urotropine, methylene blue, and salol; sedatives as bromides, liquor potassæ, and hyoscyamus; balsamics as sandal-wood oil and winter green oil should be given with the hot sitz bath, and treatment of the testicle carried out if necessary with guiacol, suspension, and poultices. The irritability does not lie in the stricture itself, but is a manifestation of prostatitis, etc., and must be controlled by the above remedies before local treatment can be concontinued. If this course fails or the condition threatens life, external urethrotomy must be done at once; delay should only be to improve the patient's condition. Where relapsing epididymitis can be relieved in no other way the vas may be cut and tied. When a stricture is actually impassable (as a rule on account of its tortuosity) it calls for perineal section, but before doing this catheterisation should be attempted after reducing congestion and spasm. This is done by first giving a hot sitz bath, then distending the urethra in front of the stricture with a solution of 5 per cent. cocaine in 1 in 5000 adrenalin for five minutes and then replacing the latter with sterile olive oil. Filiform whale-bone bougies should then be passed until one enters the bladder, and then along it a small tunnelled catheter can be passed, if necessary exerting a good deal of force. Whether the tunnelled catheter is introduced or not the filiform bougie should be tied in for 24 hours when probably it will be possible to pass tunnelled catheters up to No. 10 or 12. At times strictures which respond to no other form of treatment will become passable under the influence of mild electric currents. Complications must modify both treatment and prognosis. In making a prognosis, the position of the stricture must be taken into account.

In front of the prescrotal angle it will probably contract slowly, and never be tight enough to cause retention. In this region it will be found resilient and difficult to cure without internal urethrotomy, but once dilated or cut and kept open by sounds for three or four months, the cure should be permanent. Strictures which lie deeper are more serious. If neglected they will cause retention, but if taken in time and dilated they resolve with wondrous rapidity, but afterwards will inevitably relapse, although it may take years for them to do so. Traumatic strictures contract rapidly, but gonorrhœal strictures contract slowly. For dilation to be sufficient it should admit a full-sized sound, and remain thus for three months. At times, after years of treatment, strictures of the deeper urethra may be fully cured.

[1] Med. News, July 9, 1904.

